# An innovative quantum–fuzzy paradigm for time- and context-sensitive membership: Quantive logic

**DOI:** 10.1371/journal.pone.0354504

**Published:** 2026-07-30

**Authors:** Mehmet Akif Yerlikaya, Ömer Faruk Efe, Burak Efe, Hatef Javadı, Nicola Epıcoco

**Affiliations:** 1 Bitlis Eren University, Department of Mechanical Engineering, Bitlis, Turkiye; 2 Bursa Technical University, Department of Industrial Engineering, Bursa, Turkiye; 3 Necmettin Erbakan University, Department of Industrial Engineering, Konya, Turkiye; 4 Hacettepe University, Department of Industrial Engineering, Ankara, Turkiye; 5 LUM “Giuseppe Degennaro”University, Department of Engineering, Casamassima, Italy; Chinese Academy of Sciences Academy of Mathematics and Systems Science, CHINA

## Abstract

Uncertainty in real-world decision problems is rarely static or one-dimensional: the relevance of evidence changes over time, depends on context, and is shaped by interactions between multiple criteria. Classical fuzzy logic provides a flexible scalar notion of membership, but it typically treats each criterion in isolation and does not natively encode temporal or contextual dynamics. Quantum-inspired models, on the other hand, offer rich Hilbert-space representations but are often difficult to integrate with everyday decision-making tasks. This paper proposes Quantive Logic (QL), a quantum-inspired fuzzy framework for time- and context-sensitive membership. In QL, each element is assigned a quantive membership state, represented as a vector in a complex Hilbert space. Conventional fuzzy degrees are recovered as suitable projections of this state, while phase and superposition capture interactions and context effects between criteria. We formalize how quantive membership states are initialized from classical information and updated through linear operators that model temporal evolution and contextual shifts. To illustrate the framework, we outline a multi-criteria credit-risk assessment scenario in which applicant profiles are encoded as quantive membership states and updated under changing economic conditions. This example shows how QL can refine risk judgments when interactions between criteria and scenario-dependent effects are important. Rather than competing with existing fuzzy or probabilistic models, QL is intended as a complementary layer that enriches membership representation wherever time, context, and interaction effects cannot be ignored.

## 1. Introduction

Uncertainty is a complex and multifaceted concept that lies at the heart of contemporary scientific exploration. The theoretical frameworks that range from classical logic to fuzzy logic and even quantum logic mark significant advancements in our understanding and modeling of various aspects of uncertainty. However, current methodologies still struggle to represent uncertainty in a way that captures its richness, dynamism, and interactivity. While various logical frameworks, including temporal fuzzy systems and type-2 models, have sought to incorporate aspects of time, higher-order uncertainty, or contextual nuance, the simultaneous integration of language, meaning, phase, and multi-criteria interactions remains largely underexplored. Classical logic provides a precise binary treatment of truth, which is not tailored to represent graded uncertainty levels or probabilistic ambiguity found in complex systems. Fuzzy logic [1], on the other hand, quantifies uncertainty by defining membership degrees as continuous variables within the [0,1] range. This method, which utilizes linguistic terms, fuzzy rules, and graded membership, has led to significant practical advancements and is widely used in applications such as control engineering, expert systems, multi-criteria decision making, and data mining and in several fields (see, for instance, the contributions in Perez-Ornelas *et al.* [2] for image processing, Dotoli and Epicoco [3] for supply chain network design, Epicoco and Massaro [4] for continuous improvements, Shen [5] for production scheduling, Sabripoor *et al.* [6], for organ transplant operation, Salehi *et al.* [7] for process mining, and so on).

Though extensions such as temporal fuzzy logic [8], dynamic fuzzy systems [9], and type-2 fuzzy sets partially incorporate time and higher-order uncertainty, explicit modeling of phase interactions and superpositions—common in quantum frameworks—remains relatively rare. Consequently, it is limited in real-world situations where meaning and uncertainty typically exist in a multidimensional space and evolve in a dynamic and interactive manner. In contrast, quantum mechanics and quantum logic approach uncertainty from a fundamentally different angle, employing probability amplitudes and measurement operators. Quantum logic, characterized by its orthomodular lattice structures, Hilbert space representations, projectors, and principles of superposition, conveys uncertainty in a way that transcends simple scalar values—utilizing vectorial structures and phase effects. This perspective takes into account state vectors. This approach, which considers state vectors and their correlations (entanglement), provides a more expressive power than classical and fuzzy logic. Quantum logic has primarily evolved to formalize physical systems. While its application to cognitive and semantic uncertainty is emerging—for example in quantum cognition studies—the development of comprehensive frameworks that explicitly address linguistic ambiguity, contextual influences on decision-making, or complex interactions between conceptual domains remains relatively limited. At this point, the new approach we call Quantive Logic seeks to address this gap in the literature by thoroughly modeling the multidimensional, context-sensitive, and temporally evolving aspects of uncertainty. Quantive Logic seeks to synthesize elements from classical, fuzzy, and quantum-inspired frameworks, offering a complementary perspective that represents meaning and membership through multidimensional state vectors in a Hilbert space, enriched by explicit phase, superposition, and contextual operators. This method replaces a single membership degree with a complex membership structure that can exist in superposition, enhanced by phase differences and interactions.

The Quantive Logic formalism approach is one that unites time, context, and the dependency of data into a single mathematical model. As a result, situations that we come across on a daily basis – for example, a semantic domain of a word or a concept, decision making with a user in a certain mental state, or the branding of a single product in several segments of the market – can be modeled quite easily and more comprehensively. Quantive Logic can also fit robustly into the existing machine learning techniques, which have been trained using empirical data and are ready for real-time data input. This flexibility creates new opportunities in both fundamental science and industry applications, including such as, word sense disambiguation, polysemy, contextual ambiguity, sentiment, user behavior, and medical diagnosis assistive systems.

In this study, we provide here the basic theoretical basis of the Quantive Logic approach and describe how some quantum mechanical structures can be adapted in view of the representation of meaning and membership. Then we will set our framework within the perspective given by current literature by discussing how several known conceptual bridges between fuzzy and quantum logics can extend within the Quantive Logic paradigm. In conclusion, we will show examples of either simulation results or possible real scenarios showing how theoretical innovations of Quantive Logic can look in real life. By mathematically representing the dynamic, multidimensional, interactive, and context-sensitive nature of uncertainty, Quantive Logic provides an alternative modeling approach that could enrich existing analyses in machine learning, semantics, cognitive sciences, economics, and psychology. Thus, quantitative logic is not a purely theoretical extension but also the chance for interpreting uncertainty more subtly and practically.

## 2. Literature review

### 2.1. Classical and fuzzy logic: fundamental approaches and limitations

Classical logic has been around since Aristotle and is based on a 0–1 concept of truth [10,11]. It works well for absolute truth conditions but struggles with complex uncertainties or the graded nature of meaning. Zadeh’s fuzzy logic represents uncertainty through membership degrees in the [0,1] range and formalizes notions like “approximately” or “partially” [12]. Although used in areas like control engineering, expert systems, and data mining, fuzzy logic typically limits membership to a single scalar value. Hence it doesn’t directly deal with time, context and multidimensional interactions.

### 2.2. Quantum logic and orthomodular lattices: uncertainty in Hilbert space

Quantum logic was developed from the foundations of quantum mechanics and abandons the distributive principle of classical logic, using orthomodular lattices as the basis [13,14]. Here uncertainty is represented by projectors, superposition, phase differences and entanglement in a Hilbert space framework [15,16]. While quantum logic provides a rich mathematical structure for uncertainty, it is tied to physical measurements and therefore has limited direct application to areas like linguistic ambiguity, semantics or psychology. But quantum logic shows that uncertainty can be represented in a multi-dimensional vector space with complex amplitudes [17,18]. Recent surveys like [19] have extensively mapped the landscape of quantum machine learning, emphasizing how Hilbert space representations of uncertainty using superposition and entanglement are now operationalized in diverse applications ranging from optimization to cryptography.

### 2.3. Fuzzy quantum logic, effect algebras, and hybrid structures

Fuzzy logic and quantum logic are being merged in the works of Prugovečki [20], Pykacz [21], Goguen [22] and the Slovak group. Pykacz [21]’s “Fuzzy Quantum Logic I” is a good starting point, it’s a historical overview of the approaches of the Slovak and Italian groups and of the conceptual problems of the field. These papers show that while applying fuzzy set theory to quantum logic reveals fundamental problems, the field is not yet mature. Effect algebras [23–25] are flexible frameworks that try to bridge graded (fuzzy) uncertainty and quantum logical principles. The Slovak group and other authors have introduced fuzzy quantum spaces and fuzzy quantum posets, so the theoretical diversity is increasing. But the large scale practical applicability of these models is still limited. Recent implementations such as Qu et al. [26] with quantum fuzzy federated learning, Khushal and Fatima [27] with FQML for medical diagnosis, and Acampora et al. [28] with a quantum fuzzy inference engine for accelerator control, showcase how hybrid fuzzy-quantum logic is no longer purely theoretical but underpins real-world systems.

### 2.4. Recent studies examining the fuzzy-quantum relationship in depth

Schmitt et al. [29] study “On the Relation between Fuzzy and Quantum Logic” provides a thorough analysis of the similarities and differences between fuzzy logic and quantum logic. This work demonstrates that projector-based operations in quantum logic can correspond to algebraic t-norm products in fuzzy logic under certain conditions. Thus, quantum logic can serve as a tool to illuminate the semantic foundations behind fuzzy norms and their combinations. In recent years, research has expanded into areas like artificial intelligence, natural language processing, and semantics, using quantum-inspired models. For example, Coecke et al. [30] have combined distributional semantics with quantum formalizations to represent meaning in vector spaces. Moretti [31] and Poggiolesi [32] have deepened the axiomatic and algebraic foundations at the intersection of fuzzy logic and quantum structures. Gudder [33] has explored the relationships between quantum sets and fuzzy sets, indicating that fuzzy quantum logic can be extended to new-generation applications.

### 2.5. Positioning of quantive logic and ıts specific contribution

The reviewed literature shows that classical fuzzy logic, quantum logic, fuzzy quantum logic, effect-algebraic structures, and recent quantum-inspired fuzzy systems address different aspects of uncertainty, but they do not solve the same modelling problem. Classical fuzzy logic provides a practical scalar representation of graded membership through values in [0,1], which has made it widely applicable in control, expert systems, and decision-making [1,12]. However, this scalar representation does not natively encode phase relations, contextual rotations, or interference among criteria. Quantum logic, in contrast, is grounded in Hilbert-space projectors, subspaces, and orthomodular lattice structures [13–15]. Although this provides a richer mathematical representation of uncertainty, quantum logic was originally developed for physical measurement systems and is not directly formulated as an operational decision-support layer for empirical multi-criteria membership evaluation. Previous studies on fuzzy quantum logic and related hybrid structures have established important bridges between fuzziness and quantum-logical reasoning. Early works by Prugovečki [20] and Pykacz [21] examined the compatibility between fuzzy structures and quantum logic. Similarly, effect algebras introduced by Foulis and Bennett [23] and further developed by Dvurečenskij [24] and Dvurečenskij and Pulmannová [25] provided formal tools for representing unsharp, graded, or partially defined quantum-like events. Schmitt et al. [29] also clarified mathematical relationships between fuzzy and quantum logic, particularly by showing how some fuzzy operations can be interpreted in relation to quantum-logical structures. These studies are theoretically significant, but their main focus is algebraic, foundational, or measurement-theoretic rather than the construction of an empirical, phase-sensitive, and context-updatable decision model.

Recent studies have further extended quantum-inspired and quantum-fuzzy modelling toward machine learning, inference, and practical computation. For example, quantum fuzzy inference engines have been developed for control-oriented applications [28], quantum fuzzy federated learning has been proposed for privacy-preserving intelligent information processing [26], and fuzzy quantum machine-learning logic has been explored for disease prediction [27]. In addition, broad reviews of quantum machine learning have emphasized the growing role of Hilbert-space representations, superposition, and quantum-inspired computation in artificial intelligence applications [19]. Nevertheless, these approaches generally focus on quantum computation, quantum-enhanced inference, or application-specific learning architectures. They do not explicitly redefine fuzzy membership itself as a phase-sensitive, time-sensitive, and context-updatable complex state for multi-criteria decision analysis. Quantive Logic is proposed to address this specific gap. Its contribution is not merely the use of a Hilbert-space representation, since such representations are already well established in quantum logic and quantum-inspired modelling [13,17,18]. Rather, the novelty of Quantive Logic lies in transforming fuzzy membership evaluation into an operational complex-valued membership state. In this representation, the magnitude component preserves the intuitive meaning of classical fuzzy membership strength, while the phase component encodes contextual orientation, criterion alignment, opposition, or latent interaction. Thus, two alternatives may have similar scalar fuzzy membership values but lead to different decision outcomes because their phase configurations differ. This property allows Quantive Logic to capture constructive and destructive interference effects that ordinary scalar membership functions cannot directly represent without introducing additional interaction terms or ad hoc correction factors.

In this sense, Quantive Logic differs from classical fuzzy logic, fuzzy quantum logic, effect-algebraic approaches, quantum cognition, and quantum-inspired machine-learning models in its operational purpose. Classical fuzzy logic is mainly scalar and rule-based [1,12]; fuzzy quantum logic and effect algebras are primarily foundational and algebraic [21,23,25]; quantum cognition uses quantum probability to explain cognitive and behavioural phenomena [17,18]; and quantum-inspired learning models usually focus on computational implementation or prediction [26,28]. Quantive Logic, by contrast, defines a decision-oriented modelling pipeline: concept dimensions are specified, magnitude and phase values are assigned or learned, quantive membership states are normalized, archetype or condition vectors are constructed, contextual or temporal operators update the state, and final membership projections are calculated. Therefore, Quantive Logic fills a distinct gap in the literature by integrating four elements within a single operational framework:

(i) complex-valued membership states,
(ii) phase-dependent interference among criteria,
(iii) time- and context-dependent updating through linear or unitary operators, and
(iv) decision-oriented projection onto interpretable condition vectors.

This combination enables Quantive Logic to serve as a complementary layer to existing fuzzy, probabilistic, and quantum-inspired models rather than as a replacement for them. It is particularly relevant when scalar membership degrees are insufficient to represent contextual contradiction, hidden alignment, temporal shifts, or non-additive criterion interactions. Table 1 summarizes the technical positioning of Quantive Logic relative to the main related frameworks and clarifies the specific modelling gap addressed by the proposed approach.

**Table 1:** comparison of different logical approaches.

Dimension	Classical Logic	Classical Fuzzy Logic	Quantum Logic	Fuzzy Quantum Logic/ Effect Algebras	Quantum Fuzzy Inference/ QML Models	Quantive Logic
Basic representation of truth or membership	Binary truth values	Scalar membership degree in [0,1]	Subspaces, projectors, and Hilbert-space propositions	Graded or effect-based structures connecting fuzzy and quantum formalisms	Fuzzy inference or learning implemented through quantum or quantum-inspired mechanisms	Complex-valued membership state vector
Core mathematical object	Boolean proposition	Membership function	Projection operator/ orthomodular lattice	Effect algebra, fuzzy quantum space, fuzzy quantum poset	Quantum circuits, fuzzy rules, quantum learning operators	Quantive membership state and archetype vectors
Treatment of phase	Not included	Not included	Phase exists in the underlying Hilbert-space formalism	Usually treated at a foundational or algebraic level	May appear through quantum computational representation	Explicitly used as a decision-relevant membership component
Treatment of criterion interaction	Logical conjunction/disjunction	Usually modeled through t-norms, rules, or aggregation operators	Non-commutativity and interference are native to the formalism	Interaction is theoretically expressible through generalized quantum structures	Interaction may be encoded in quantum circuits or fuzzy inference structures	Phase-dependent constructive/destructive interference among criteria
Context sensitivity	Not represented	Possible through changing rules or membership functions	Measurement context is central but physically motivated	Mainly theoretical or measurement-oriented	Usually task-specific learning or inference context	Explicit context operators update membership states
Time sensitivity	Static	Usually static unless extended by dynamic fuzzy systems	Time evolution possible through unitary operators	Not usually formulated as empirical decision updating	May be handled through model training or dynamic data streams	Time/context evolution is part of the membership-state formulation
Empirical decision pipeline	Rule-based logical decision	Fuzzy rule base or fuzzy aggregation	Not primarily designed for ordinary decision-support pipelines	Mostly foundational and algebraic	Application-specific computational pipeline	Magnitude-phase encoding, normalization, projection, context update, and decision threshold
Interpretability focus	High but binary	High through linguistic variables	Low for non-physics users	Often abstract and mathematical	Depends on implementation	Interpretable through magnitude, phase alignment, interference, and archetype projection
Main limitation addressed by QL	Cannot represent graded uncertainty	Cannot directly represent phase-sensitive contextual interaction	Not designed as a practical fuzzy membership model	Limited operational decision pipeline	Often focuses on computation rather than membership semantics	Provides phase-sensitive, context-updatable fuzzy membership representation

## 3. The theoretical foundations and model of the quantive logic approach

### 3.1. Motivation and conceptual overview

Membership in classical logic and fuzzy logic is generally expressed through static numerical values. Membership in classical logic is binary (0 or 1), while in fuzzy logic, membership is a scalar in [0,1]. Although fuzzy logic generalizes crisp (Boolean) sets to sets membership degrees, the fuzzy logic has little expressive power to work with time-dependent, context-, and inherently multidimensional concepts. Quantum logic arose from attempts to comprehend the logical framework foundational to quantum mechanics. It treats Hilbert spaces and projection operators as the representations of propositions and uncertainties. Quantum logic can describe probabilities beyond classical or fuzzy logic implications (e.g., interference, entanglement) via complex amplitudes. Quantive Logic is an attempt to bring these ideas together. Logically, it creates a space of concepts and their memberships not in terms of scalars frozen in time, but in terms of vector states living in a Hilbert space. Such a vector-based model supports superposition of conceptual meanings, membership states change over time and context, and also phase effects [17]. Quantive Logic makes a more energetic and varied model than existing frameworks by running it this way.

In this new logical prism, we find a new concept of membership — Quantive Membership — just as fuzzy logic has fuzzy membership functions. Quantive Membership gives each element a vector state that encodes its relationship to several concepts at once. We have not one complex number valued membership (scalar) instead we have a complex amplitude structure that can reflect context effects, time effects and interaction effects.

### 3.2. Mathematical foundations

#### 3.2.1. Hilbert Space and Basic Notation.

Let H be a complex Hilbert space. Unless stated otherwise, we assume H to be separable. An inner product ⟨·∣·⟩:H×H→C is linear in the second argument and conjugate-linear in the first. For any |ψ⟩∈H, we denote its norm by $∥\psi ∥=\sqrt{\langle \psi |\psi \rangle }$ [31].

- Objects and Concepts: We have a universe Ω of elements or instances we wish to describe. We have a family of concepts ${\left\{C_{i}\right\}}_{i\in I}$, where I is an index set that may be finite or countably infinite.
- Concept Vectors: We represent each concept $C_{i}$ by a normalized vector $\left|w_{i}\right\rangle \in H$ with $\left\langle w_{i}|w_{i}\right\rangle =\text{1}$. The set $\left\{\left|w_{i}\right\rangle :i\in I\right\}$ need not form an orthonormal basis. In fact, overlaps $\left\langle w_{i}|w_{j}\right\rangle$ for i≠j represent conceptual correlations.

*Definition 3.1 (Concept Representation):*

Let $C={\left\{C_{i}\right\}}_{i\in I}$ be a set of concepts. A concept representation system is a map $C_{i}\mapsto \left|w_{i}\right\rangle \in H$ such that each $\left|w_{i}\right\rangle$ is a unit vector. The pair $\left(H,\left\{\left|w_{i}\right\rangle \right\}\right)$ is called a conceptual Hilbert system.

#### 3.2.2. Quantive Membership States and Functions.

In fuzzy logic, a membership function $\mu_{M}(x)$ assigns a real number in [0,1] to an element x and a concept M.  [1]. In Quantive Logic, we introduce Quantive Membership to generalize this idea. Each element x∈Ω is mapped to a vector in Hilbert space, encoding a multidimensional amplitude structure rather than a single scalar: Each element x∈Ω is mapped to a vector |μ(x)⟩∈H.

*Definition 3.2 (Quantive Membership Function):*

A Quantive Membership function is a map and is expressed as in Equation 1:


$$\begin{array}{c} \;\mu :\Omega \to H,\;x\mapsto \left|\mu (x)\right\rangle \end{array}$$ (1)

For each x,|μ(x)⟩ is called the Quantive membership state of x. To interpret the membership of x to a specific concept $C_{i}$, we project |μ(x)⟩ onto $\left|w_{i}\right\rangle$. This means that instead of simply assigning a number like 0.6 to indicate that x belongs to a concept, we map x to a richer state |μ(x)⟩, which encodes its potential associations to all concepts simultaneously. It is similar to creating a ‘profile’ for x that carries information about how x is related to different concepts.

*Definition 3.3 (Quantive Membership Amplitude and Degree):*

For element x and concept $C_{i}$:


$$\begin{array}{c} {\;\alpha }_{i}(x)\;\;:\;=\left\langle w_{i}|\mu (x)\right\rangle \in C,\; \end{array}$$ (2)

called the membership amplitude, and the corresponding membership degree is expressed as:


$$\begin{array}{c} {\;m}_{i}(x)\;\;:\;={\left|\alpha_{i}(x)\right|}^{\text{2}}\in [\text{0},\infty )\; \end{array}$$ (3)

To ensure normalization, we often impose conditions that bound these degrees. Typically, we may normalize |μ(x)⟩ such that $\sum_{i}\;m_{i}(x)=\text{1}$ (if desired), making the distribution $\left\{m_{i}(x)\right\}$ analogous to a probability distribution over concepts. Here, $\alpha_{i}(x)$ measures how strongly x aligns with concept $C_{i}-$ like projecting x ‘s state onto the direction representing $C_{i}$. Then $m_{i}(x)$ quantifies this on a scale of intensity, telling us how much x ‘belongs’ to concept $C_{i}$. This is similar to fuzzy logic membership but built on a quantum-inspired structure, allowing for richer overlaps and interference effects between concepts.

*Remark:* If no normalization is imposed, we can interpret ratios or relative comparisons. Normalization is convenient for probabilistic interpretations.

#### 3.2.3. Superposition, context, and time evolution.

The vector |μ(x)⟩ can be expressed as:


$$\begin{array}{c} \left|\mu (x)\right\rangle =\sum_{i\in I}\;\beta_{i}(x)\left|w_{i}\right\rangle ,\; \end{array}$$ (4)

with $\beta_{i}(x)\in C$. This represents a superposition of conceptual components, each carrying an amplitude that may have a phase factor.

- Context: Contextual information can be encoded as a unitary or bounded linear operator U(C):H→H that depends on the context C. Then:

$$\begin{array}{c} \;\left|\mu (x;C)\right\rangle \;:=U(C)\left|\mu (x)\right\rangle ,\; \end{array}$$ (5)

adjusting the state according to contextual conditions.

- Time Evolution: Similarly, to model temporal changes, we consider a strongly continuous one parameter unitary group {U(t):t∈R} with U(0)=I, representing time evolution:

$$\begin{array}{c} \;\left|\mu (x;t)\right\rangle \;:=U(t)\left|\mu (x;\text{0})\right\rangle \; \end{array}$$ (6)

This structure allows Quantive Logic states to dynamically change over time and context, extending fuzzy logic from static membership functions to time- and context-sensitive vector states.

One of the key advantages of incorporating phase factors into |μ(x)⟩ is the ability to model phase dependent interference effects [34]. Unlike static fuzzy logic, where components are scalar and additive, Quantive Logic introduces phase-sensitive correlations, which can modify the resulting membership degrees through constructive or destructive interference. For example, consider two conceptual components $\left|w_{\text{1}}\right\rangle$ and $\left|w_{\text{2}}\right\rangle$, with amplitudes $\beta_{\text{1}}(x)=r_{\text{1}}e^{i\theta_{\text{1}}}$ and $\beta_{\text{2}}(x)=r_{\text{2}}e^{i\theta_{\text{2}}}$, respectively. Their combined contribution to the membership degree is expressed as follows:


$$\begin{array}{c} \;|\langle w|\mu (x;C)\rangle {|}^{\text{2}}={\left|r_{\text{1}}e^{i\theta_{\text{1}}}+r_{\text{2}}e^{i\theta_{\text{2}}}\right|}^{\text{2}}=r_{\text{1}}^{\text{2}}+r_{\text{2}}^{\text{2}}+\text{2}r_{\text{1}}r_{\text{2}}\text{cos}\left(\theta_{\text{1}}-\theta_{\text{2}}\right)\; \end{array}$$ (7)

Here, the phase difference $\theta_{\text{1}}-\theta_{\text{2}}$ determines whether the contributions from $\left|w_{\text{1}}\right\rangle$ and $\left|w_{\text{2}}\right\rangle$ interfere constructively $(\text{cos}\left(\theta_{\text{1}}-\theta_{\text{2}}\right)>\text{0})\;$ or destructively $(\text{cos}\left(\theta_{\text{1}}-\theta_{\text{2}}\right)<\text{0}\;$). This interference mechanism enables Quantive Logic to dynamically adjust the membership degrees based on both the context and the relationships between conceptual components. Such adjustments are particularly useful in decision-making scenarios where criteria interact in non-additive ways, providing a more nuanced representation of their interplay.

#### 3.2.4. Observables and conceptual measurements.

In quantum mechanics, measuring an observable associated with a projection P yields probabilities given by ⟨ψ|P|ψ⟩. In Quantive logic, checking membership to concept $C_{i}$ is analogous to projecting |μ(x)⟩ onto $\left|w_{i}\right\rangle$:


$$\begin{array}{c} {\;P}_{i}=\left|w_{i}\right\rangle \left\langle w_{i}\right|\; \end{array}$$ (8)

The membership degree to $C_{i}$ is expressed as:


$$\begin{array}{c} \;m_{i}(x)=\left\langle \mu (x)\left|P_{i}\right|\mu (x)\right\rangle ={\left|\left\langle w_{i}|\mu (x)\right\rangle \right|}^{\text{2}}\; \end{array}$$ (9)

**Fig 1:**
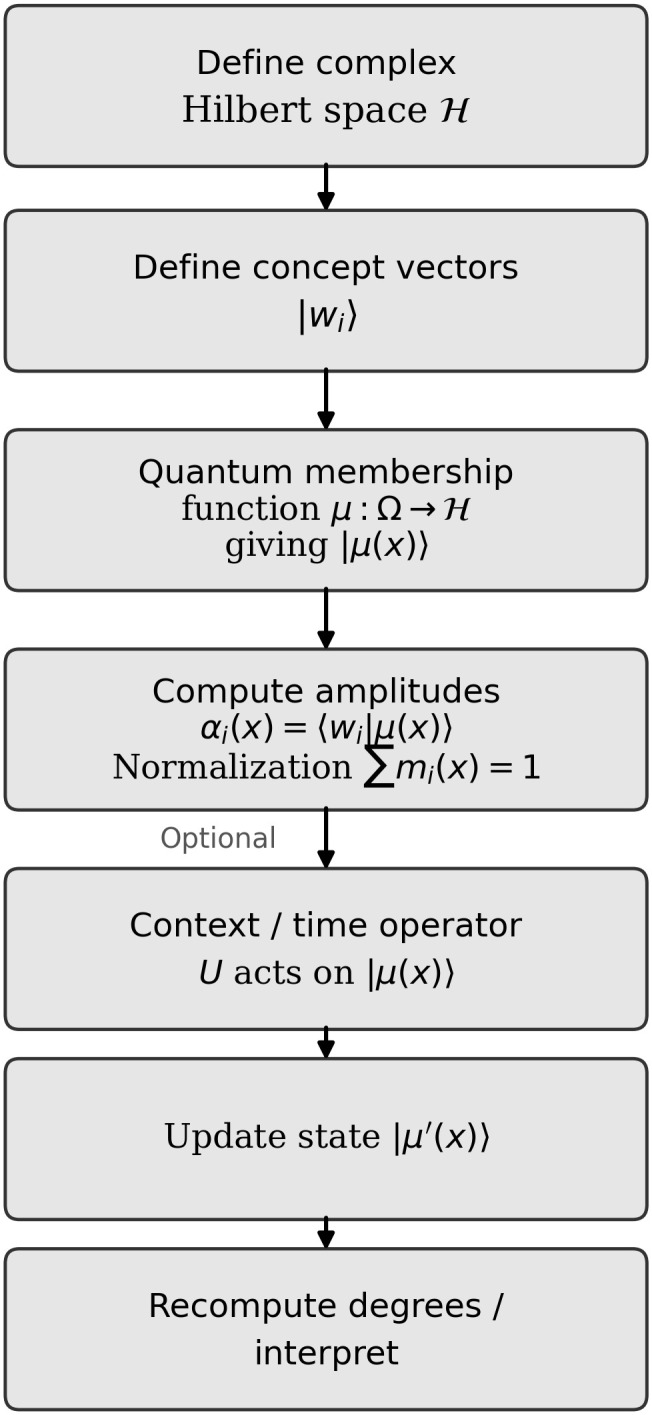
Theoretical pipeline of Quantive Logic.

This parallels quantum probability, but here it represents conceptual membership. Non-commuting projections represent concepts that cannot be jointly defined without interference effects, akin to non-commuting observables in quantum mechanics. Flowchart in Fig 1, summarizes the fundamental steps of Quantive Logic. It begins by defining a complex Hilbert space H and an orthonormal set of concept vectors $\left|w_{i}\right\rangle$ corresponding to the domain’s conceptual dimensions. The Quantive membership function μ:Ω→H then maps each object x to a membership state |μ(x)⟩. Membership amplitudes $\alpha_{i}(x)=\left\langle w_{i}|\mu (x)\right\rangle$ are computed, with optional normalization to ensure $\sum_{i}\;m_{i}(x)=\text{1}$. If external context or temporal evolution is considered, a unitary operator U acts on |μ(x)⟩, yielding an updated state $\left|\mu^{\prime }(x)\right\rangle$. The updated membership degrees are recomputed, enabling interpretation and decision-making based on the Quantive logic framework.

### 3.3. Fundamental results and proofs

We now establish key theorems that guarantee the well-definedness, expressive power, and consistency of Quantive Logic. We provide detailed proofs to ensure mathematical rigor. For functional analytic details see Kadison and Ringrose (Kadison & Ringrose, 1997) or for Stone’s theorem see Reed and Simon [35].

#### 3.3.1. Existence and constructibility of quantive membership states.

Given an indexed family of concepts ${\left\{C_{i}\right\}}_{\in I}$, each represented by a normalized vector $\left|w_{i}\right\rangle \in H$, and given any function $\mu :\Omega \to {\text{𝓁}}^{\text{2}}(I)$ (where ${\text{𝓁}}^{\text{2}}(I)$ is the space of square-summable complex sequences), there exists a Quantive membership state |μ(x)⟩∈H for each x∈Ω defined as follows:


$$\begin{array}{c} |\mu (x)\rangle :=\sum_{i\in I}\;\beta_{i}(x)\left|w_{i}\right\rangle , \end{array}$$ (10)

where $\beta_{i}(x)$ are the coordinates given by μ(x).

**Proof:**

1 Well-definedness: Let $\mu (x)={\left(\beta_{i}(x)\right)}_{i\in I}$ be a sequence with $\sum_{i}\;{\left|\beta_{i}(x)\right|}^{\text{2}}<\infty$. Since $\left|w_{i}\right\rangle$ are unit vectors, the series $\sum_{i}\;∥\beta_{i}(x)\left|w_{i}\right\rangle ∥$ converges absolutely or at least is bounded by a constant times $\sqrt{\sum_{i}\;{\left|\beta_{i}(x)\right|}^{\text{2}}}$.
2 Closure under linear combinations: The set $\left\{\left|w_{i}\right\rangle \right\}$ spans a (not necessarily orthonormal) set of vectors. If it is not a basis, we consider its closed linear span $\overset{―}{\text{span}}\left\{\left|w_{i}\right\rangle \right\}$. By assumption, |μ(x)⟩ lies in this closed span, since it is a limit of finite sums.
3 Existence: The vector |μ(x)⟩ exists by the completeness of H. Therefore, every element x can be assigned a Quantive membership state.

This proves that Quantive Logic states can always be constructed to represent arbitrary sets of amplitudes and concepts.

#### 3.3.2. Reduction to fuzzy logic in the orthogonal, real, positive case.

Suppose ${\left\{\left|w_{i}\right\rangle \right\}}_{i\in I}$ is an orthonormal set. If all amplitudes $\beta_{i}(x)$ are chosen real and nonnegative and the vectors are orthonormal, then for each concept $C_{i}$, we have $m_{i}(x)=$ ${\left|\beta_{i}(x)\right|}^{\text{2}}\in [\text{0},\text{1}]$ and $\sum_{i}\;m_{i}(x)=\text{1}$. In this case, Quantive membership reduces to a standard fuzzy membership function.

**Proof:**

1 Orthogonality: If $\left\langle w_{i}|w_{j}\right\rangle =\delta_{ij}$, then:

$$\begin{array}{c} m_{i}(x)={\left|\left\langle w_{i}|\mu (x)\right\rangle \right|}^{\text{2}}={\left|\beta_{i}(x)\right|}^{\text{2}} \end{array}$$ (11)

2 Normalization: Choose $\beta_{i}(x)$ such that $\sum_{i}\;{\left|\beta_{i}(x)\right|}^{\text{2}}=\text{1}$. Thus $\left\{m_{i}(x)\right\}$ forms a probability distribution. This matches the fuzzy approach with membership in [0,1].
3 No interference: Since no complex phases or non-orthogonal overlaps exist, no quantum-like interference occurs. Each concept acts like a distinct “crisp” dimension. Hence the model collapses to a standard fuzzy logic scenario.

#### 3.3.3. Quantum-like interference and increased expressiveness.

Quantive Logic strictly generalizes fuzzy logic. That is, there exist configurations of concept vectors and Quantive Logic states for which no scalar-based membership model (classical or fuzzy) can replicate the observed conceptual interference patterns.

**Proof:**

1 Consider two concepts $C_{\text{1}}$ and $C_{\text{2}}$ represented by non-orthogonal vectors $\left|w_{\text{1}}\right\rangle$ and $\left|w_{\text{2}}\right\rangle$ with $\left\langle w_{\text{1}}|w_{\text{2}}\right\rangle \text{≠}\text{0}$.
2 Define:

$$\begin{array}{c} \;|\mu (x)\rangle =\frac{\text{1}}{\sqrt{\text{2}}}\left(\left|w_{\text{1}}\right\rangle +e^{i\theta }\left|w_{\text{2}}\right\rangle \right) \end{array}$$ (12)

with θ∈R. Then it holds:


$$\begin{array}{c} {\;m}_{\text{1}}(x)={\left|\left\langle w_{\text{1}}|\mu (x)\right\rangle \right|}^{\text{2}}=\frac{\text{1}}{\text{2}},\;m_{\text{2}}(x)={\left|\left\langle w_{\text{2}}|\mu (x)\right\rangle \right|}^{\text{2}}=\frac{\text{1}}{\text{2}} \end{array}$$ (13)

3 Now define a new “concept” represented by $|u\rangle =a\left|w_{\text{1}}\right\rangle +b\left|w_{\text{2}}\right\rangle$, with a,b∈C and ∥u∥= 1. The membership probability for |u⟩ is expressed as:

$$\begin{array}{c} {\;m}_{u}(x)=|\langle u|\mu (x)\rangle {|}^{\text{2}}={\left|a\frac{\text{1}}{\sqrt{\text{2}}}+b\frac{e^{i\theta }}{\sqrt{\text{2}}}\right|}^{\text{2}\;} \end{array}$$ (14)

4 By adjusting θ, we produce constructive or destructive interference. For certain values of θ, $m_{u}(x)$ can differ significantly from any convex combination of $\left\{m_{\text{1}}(x),m_{\text{2}}(x)\right\}$. No scalarbased model can reproduce a phase-dependent shift in membership without altering base memberships $m_{\text{1}}(x),m_{\text{2}}(x)$. Expanding effect of θ:

$$\begin{array}{c} \;m_{u}(x)=\frac{\text{1}}{\text{2}}{\left|a+be^{i\theta }\right|}^{\text{2}} \end{array}$$ (15)

- Using the Euler formula $e^{i\theta }=\text{cos}\theta +i\text{sin}\theta$, the real and imaginary components of the interference are expressed as:

$$\begin{array}{c} \;m_{u}(x)=\frac{\text{1}}{\text{2}}\left[(a+\text{bcos}\theta {)}^{\text{2}}+(\text{bsin}\theta {)}^{\text{2}}\right] \end{array}$$ (16)

This reveals that θ can create constructive interference (when cos θ > 0) or destructive interference (when cos θ < 0) between a and b. The interference depends directly on θ, making m_u_(x) highly sensitive to phase relationships. Special Cases for θ:

θ= 0: Maximum constructive interference. The terms a and b add in-phase as:


$$\begin{array}{c} m_{u}(x)=\frac{\text{1}}{\text{2}}|a+b{|}^{\text{2}} \end{array}$$ (17)

θ= π: Maximum destructive interference. The terms a and b are out of phase as follows:


$$\begin{array}{c} \;m_{u}(x)=\frac{\text{1}}{\text{2}}|a-b{|}^{\text{2}}\; \end{array}$$ (18)

*θ* = π/2: Purely imaginary phase shift. The interference is orthogonal, and the terms interact via their imaginary components:


$$\begin{array}{c} \;m_{u}(x)=\frac{\text{1}}{\text{2}}\left[a^{\text{2}}+b^{\text{2}}\right] \end{array}$$ (19)

- For any fixed $\theta ,m_{u}(x)$ can significantly differ from any convex combination of $m_{\text{1}}(x)$ and $m_{\text{2}}(x)$. For example:

$$\begin{array}{c} m_{u}(x)=\frac{\text{1}}{\text{2}}{\left|a+be^{i\theta }\right|}^{\text{2}} \end{array}$$ (20)

includes cross terms from a and b, which scalar-based models cannot reproduce without altering $m_{\text{1}}(x)$ or $m_{\text{2}}(x)$. This behavior, driven by θ, is unique to Quantive Logic and cannot be replicated by classical or fuzzy logic. By allowing phase-dependent interference, Quantive Logic introduces an expressive power that scalar-based models, including fuzzy logic, lack. This interference behavior, controlled by θ, demonstrates the strictly greater generality and expressiveness of Quantive Logic.

5 Thus, Quantive logic exhibits interference effects analogous to quantum phenomena. This cannot be mimicked by fuzzy logic, proving Quantive logic’s strictly greater expressiveness.

#### 3.3.4. Contextual and temporal adaptability.

Given a Quantive Membership function μ:Ω→H and a continuous unitary representation of contexts C and times t, we can generate context- and time-dependent membership states |μ(x;C,t)⟩=U(C,t)|μ(x;0)⟩, ensuring continuity and consistency over a parameter space.

**Proof Outline:**

1 Assume {U(C,t):C∈C,t∈R} is a strong continuous unitary group acting on H. By Stone’s theorem, U(C,t)=exp(−iH(C)t) for some self-adjoint operator H(C) depending on context C.
2 Define:

$$\begin{array}{c} |\mu (x;C,t)\rangle :=U(C,t)\left|\mu (x;\text{0})\right\rangle \end{array}$$ (21)

This is well-defined since U(C,t) is unitary and bounded.

3 The continuity in t and the smooth variation with respect to context C ensure stable and welldefined evolutions. Membership degrees become time- and context-dependent:

$$\begin{array}{c} {\;m}_{i}(x;C,t)={\left|\left\langle w_{i}\right|U(C,t)\right|\mu (x;\text{0})\rangle }^{\text{2}} \end{array}$$ (22)

providing a continuous and dynamic adaptation mechanism.

#### 3.3.5. Boundedness of quantive membership degrees.

For any normalized Quantive membership state |μ(x)⟩ and any normalized condition vector $\left|w_{r}\right\rangle$, the Quantive membership degree is bounded within the unit interval:


$$\begin{array}{c} \text{0}\leq {\left|\left\langle w_{r}|\mu (x)\right\rangle \right|}^{\text{2}}\leq \text{1}. \end{array}$$ (23)

**Proof Outline:**

Since both |μ(x)⟩ and $\left|w_{r}\right\rangle$, are assumed to be normalized, we have


$$\begin{array}{c} \|\mu (x)\|=\text{1} \end{array}$$ (24)

And


$$\begin{array}{c} \left\|w_{r}\right\|=\text{1} \end{array}$$ (25)

By the Cauchy-Schwarz inequality,


$$\begin{array}{c} \left|\left(w_{r}|\mu (x)\right)\right|\leq \left\|w_{r}\right\|\left\|\mu (x)\right\|. \end{array}$$ (26)

Thus,


$$\begin{array}{c} \left|\left(w_{r}|\mu (x)\right)\right|\leq \text{1}. \end{array}$$ (27)

Squaring both sides gives


$$\begin{array}{c} {\left|\left\langle w_{r}|\mu (x)\right\rangle \right|}^{\text{2}}\leq \text{1}. \end{array}$$ (28)

Since squared magnitudes are always non-negative, it follows that


$$\begin{array}{c} \text{0}\leq {\left|\left\langle w_{r}|\mu (x)\right\rangle \right|}^{\text{2}}\leq \text{1}. \end{array}$$ (29)

This result ensures that Quantive membership degrees remain interpretable as bounded membership or probability-like values and cannot grow without control.

#### 3.3.6. Phase inactivity under zero magnitude.

If the magnitude of a criterion is zero, then the corresponding phase has no effect on the Quantive membership projection. Let the complex contribution of criterion i be written as


$$\begin{array}{c} a_{i}=\rho_{i}e^{j\phi_{i}} \end{array}$$ (30)

where $\rho_{i}\;$is the magnitude and $\phi_{i}$ is the phase. If:


$$\begin{array}{c} \rho_{i}=\text{0}, \end{array}$$

then:


$$\begin{array}{c} a_{i}=\text{0}\cdot e^{j\phi_{i}}=\text{0}. \end{array}$$

Therefore, the contribution of criterion i disappears from the Quantive membership state regardless of the value of $\phi_{i}$.

**Proof Outline:**

For any phase angle $\phi_{i}$,


$$\begin{array}{c} e^{j\phi_{i}} \end{array}$$

has unit modulus. However, when it is multiplied by zero magnitude, the resulting complex amplitude becomes zero:


$$\begin{array}{c} \text{0}\cdot e^{j\phi_{i}}=\text{0} \end{array}$$

Thus, the corresponding term contributes neither to the real part nor to the imaginary part of the membership projection. Consequently, phase cannot independently create a membership or risk contribution in the absence of observed criterion magnitude. This result is important for interpretability and robustness. It shows that phase is not an unrestricted tuning parameter: it can modify the orientation of an existing signal, but it cannot generate evidence when the criterion magnitude is zero.

#### 3.3.7. Phase sensitivity of membership projection.

Quantive membership projections are sensitive to phase differences between criteria. Consider two conceptual components with magnitudes $\rho_{i}$ and $\rho_{j}$ and phases $\phi_{i}$ and $\phi_{i}$. Their combined squared magnitude contains an interference term of the form


$$\begin{array}{c} \text{2}\rho_{i}\rho_{j}\text{cos}\left(\phi_{i}-\phi_{j}\right) \end{array}$$ (31)

**Proof Outline:**

Let two complex components be defined as


$$\begin{array}{c} a_{i}=\rho_{i}e^{j\phi_{i}}\text{\textasciitilde{}and} \end{array}$$ (32)

$$\begin{array}{c} a_{j}=\rho_{j}e^{j\phi j} \end{array}$$

The squared magnitude of their combined contribution is


$$\begin{array}{c} {\left|a_{i}+a_{j}\right|}^{\text{2}} \end{array}$$ (33)

Expanding this expression gives


$$\begin{array}{c} {\left|a_{i}+a_{j}\right|}^{\text{2}}=\left(\rho_{i}e^{j\phi_{i}}+\rho_{j}e^{j\phi_{j}}\right)\left(\rho_{i}e^{-j\phi_{i}}+\rho_{j}e^{-j\phi_{j}}\right) \end{array}$$ (34)

Therefore,


$$\begin{array}{c} {\left|a_{i}+a_{j}\right|}^{\text{2}}=\rho_{i}^{\text{2}}+\rho_{j}^{\text{2}}+\rho_{i}\rho_{j}e^{j\left(\phi_{i}-\phi_{j}\right)}+\rho_{i}\rho_{j}e^{-j\left(\phi_{i}-\phi_{j}\right)} \end{array}$$ (35)

Using Euler’s identity, this becomes


$$\begin{array}{c} {\left|a_{i}+a_{j}\right|}^{\text{2}}=\rho_{i}^{\text{2}}+\rho_{j}^{\text{2}}+\text{2}\rho_{i}\rho_{j}\text{cos}\left(\phi_{i}-\phi_{j}\right) \end{array}$$ (36)

The final term shows that the membership projection changes according to the phase difference. If


$$\begin{array}{c} \text{cos}\left(\phi_{i}-\phi_{j}\right)>\text{0} \end{array}$$

the interference is constructive and the combined contribution increases. If


$$\begin{array}{c} \text{cos}\left(\phi_{i}-\phi_{j}\right)<\text{0} \end{array}$$

the interference is destructive and the combined contribution decreases. If


$$\begin{array}{c} \text{cos}\left(\phi_{i}-\phi_{j}\right)=\text{0} \end{array}$$

the two components are orthogonal in phase and the interference term vanishes.

This proposition formalizes why phase information changes the behaviour of Quantive Logic relative to scalar fuzzy membership models. Unlike scalar fuzzy membership models, where only the magnitude of membership is considered, Quantive Logic also incorporates phase differences between criteria. These phase differences generate constructive, destructive, or neutral interference effects, thereby allowing two cases with similar scalar membership values to produce different projected membership degrees.

#### 3.3.8. Norm preservation under unitary context operators.

If the context operator $U_{c}$ is unitary, then it preserves the norm of the Quantive membership state:


$$\begin{array}{c} \left\|U_{c}\mu (x)\right\|=\|\mu (x)\| \end{array}$$ (37)

**Proof Outline**

Since $U_{c}$ is unitary, it satisfies


$$\begin{array}{c} U_{c}^{*}U_{c}=I \end{array}$$ (38)

where $U_{c}^{*}$ denotes the adjoint of $U_{c}$ and I is the identity operator. Then,


$$\begin{array}{c} {\left\|U_{c}\mu (x)\right\|}^{\text{2}}=\left\langle U_{c}\mu (x),U_{c}\mu (x)\right\rangle \end{array}$$ (39)

Using the definition of the adjoint operator,


$$\begin{array}{c} \left\langle U_{c}\mu (x),U_{c}\mu (x)\right\rangle =\left\langle \mu (x),U_{c}^{*}U_{c}\mu (x)\right\rangle \end{array}$$ (40)

Since $U_{c}^{*}U_{c}=I$, we obtain


$$\begin{array}{c} \left\langle \mu (x),U_{c}^{*}U_{c}\mu (x)\right\rangle =\langle \mu (x),\mu (x)\rangle \end{array}$$ (41)

Therefore,


$$\begin{array}{c} {\left\|U_{c}\mu (x)\right\|}^{\text{2}}=\|\mu (x){\|}^{\text{2}} \end{array}$$ (42)

and hence


$$\begin{array}{c} \left\|U_{c}\mu (x)\right\|=\|\mu (x)\| \end{array}$$ (43)

This result shows that a unitary context update does not arbitrarily inflate or deflate the total magnitude of the borrower state. It changes the orientation of the state in the Quantive space while preserving its norm. Therefore, any change in membership probabilities after a context update arises from altered alignment with the condition vectors, not from uncontrolled scaling of the state vector.

It is important to note that the computational cost of the proposed Quantive Logic framework depends primarily on the dimensionality of the underlying Hilbert space H, which is determined by the number of concepts or basis states used. In general, computing the membership amplitudes involves inner products $\left\langle w_{i}|\mu (x)\right\rangle$ across all i, leading to a linear scaling with the number of concepts. However, as the dimensionality increases, operations such as normalization and orthogonal projections require more computational resources, scaling approximately as O(n) for n concepts. While this remains manageable for moderately sized decision problems, very high-dimensional scenarios (hundreds or thousands of concepts) may necessitate dimensionality reduction techniques or sparse representations to maintain computational efficiency. Future work may explore leveraging quantum-inspired data structures or approximate embedding techniques to further mitigate these costs.

### 3.4. Conclusion of the theoretical foundation

The Quantive Logic framework extends classical and fuzzy logic in quantum inspired vector representations, capable of capturing super positions and phase effects. The above theorems guarantee existence, give criteria for reduction back to fuzzy logic, and introduce new phenomena (interference) that can’t be modelled with traditional or fuzzy logic only. Conceptually, this matches up with coarse approaches to classical (fuzzy) quantum logic that have been developed in the past indeed, it goes them one better, by directly providing an operational definition of quantum membership states as vectors in Hilbert space. In addition, Quantive Logic also allows for the combination of machine learning approaches since states |μ(x)⟩ and concept vectors $\left|w_{i}\right\rangle$ may be learned from empirical data similar to learned embeddings in distributional semantics or representation learning. This provides one of the primary practical benefits of this construct, uniting the theoretical rigour of quantum logic with the flexibly of fuzzy logic and contemporary data-driven methodologies.

With the evidence introduced up to this point, we have made both a mathematically sound and conceptually innovative case for Quantive Logic. Quantive Membership, theoretically grounded by its own existence of Quantive Logic, its ability to reduce to simpler fuzzy logic in special cases, its representation of interference-like phenomena which provide additional expressive power, and its dynamic changes through the dimensionalities of time and context, all assure us of its theoretical soundness. We will demonstrate through examples, simulations, and real applications, how these possible benefits of Quantive Logic become real mechanics of benefit in managing such complex, uncertain, and context sensitive phenomena in later sections.

### 3.5. Potential applications and empirical validation

Although our focus so far has been on the theoretical underpinnings of Quantive Logic, the framework can be leveraged in various practical domains. Four areas particularly stand out:

1. Natural Language Processing (NLP) and Semantics

- Contextual Meaning Shifts: Membership states ∣μ(x)⟩ can be dynamically updated to reflect linguistic context (e.g., word sense disambiguation).
  - Quantum-Like Interference: Superposition of conceptual meanings can model subtle semantic combinations more effectively than simple scalar memberships.

1. Decision Making and Cognitive Modeling

- Context-Dependent Preferences: A decision-maker’s preference is treated as a time- or context-evolving vector state.
  - Explaining Paradoxes: Quantum-inspired interference can replicate observed choice anomalies in human cognition (e.g., conjunction fallacies).

1. Recommendation Systems and User Modeling

- Evolving User States: User profiles become vectors ∣μ(u)⟩, updated by unitary operators to reflect changing preferences or contexts.
  - Enhanced Expressiveness: Phase and amplitude factors allow more nuanced representations of preferences than traditional fuzzy or purely vector-based methods.

1. Biomedical Diagnostics and Sensor Data

- Complex Criteria Integration: Different diagnostic concepts (e.g., viral vs. bacterial indicators) are stored as vectors, and patient states evolve over time.
  - Real-Time Updates: Continuous data streams can modify membership states quickly, capturing rapid changes in clinical conditions.

Empirical Validation and Practical Considerations

- Model Construction and Training: Concepts are represented as vectors ∣wi⟩ and element states ∣μ(x)⟩ can be learned from data via iterative, gradient-based methods.
- Performance Metrics: Tasks such as classification accuracy, F1 scores, or recommendation quality can be compared against established fuzzy or probabilistic baselines.
- Interpretability and Scalability: While phase and interference add expressive power, large Hilbert spaces may pose computational challenges. Research on dimensionality reduction, interpretability tools, and specialized hardware (e.g., GPU-accelerated linear algebra) can help address these issues.

In summary, Quantive Logic offers a multidimensional, context-sensitive, and dynamic approach to uncertainty modeling, with promising early results in diverse domains. Future studies will benefit from large-scale empirical testing to confirm the framework’s practical advantages over classical and fuzzy alternatives.

## 4. A realistic financial risk-assessment example

### 4.1. Overview of the scenario

Credit-risk analysts seldom rely on a single number when deciding whether to approve a loan; instead, they weigh many partially conflicting signals that arrive from heterogeneous data sources (credit bureaus, open-banking feeds, device fingerprints, macro indicators, etc.). Classical scoring systems compress those signals into a scalar “probability of default,” while fuzzy scoring systems replace the hard cut-off with a graded membership in “good” or “bad” credit classes. Both approaches, however, treat each indicator as if it acted independently and additively. In practice the signals interact: a borrower who occasionally over-utilizes credit (high C₃) may still be safe if their income is stable (C₅ with reassuring phase), yet that same utilization becomes worrying when income is seasonal (identical magnitude but a phase that is now mis-aligned). To capture such *interaction* and *context sensitivity*, we recast the decision in the Quantive-Logic framework

Conceptual space: We span a 12-dimensional complex Hilbert space $H=C^{\text{12}}$ whose orthonormal axes $C_{\text{1}},\ldots ,C_{\text{12}}$ correspond to the most common credit-risk indicators listed in Table 2 (payment history trend, delinquency recency,... macroeconomic sector outlook). Each dimension carries:
  - a magnitude in [0,1] that measures signal strength (e.g., 0.92 means the repayment record is close to ideal), and
  - a phase angle $\phi_{i}\in \left(-{\text{180}}^{\circ },{\text{180}}^{\circ }\right]$ that expresses the behavioral orientation of that signal:

- ${\text{0}}^{\circ }$ fully supportive (“pulls” towards low risk);
- $+\theta^{\circ }$ raises concern by θ degrees;
$-\theta^{\circ }$ offers reassurance by θ degrees;
- $\pm {\text{180}}^{\circ }$ directly contradicts the nominal meaning (e.g., cash savings exist yet are functionally unavailable because the borrower already pledged them as collateral elsewhere).

2 Condition vectors: We will shortly derive three non-orthogonal condition vectors:

- $\left|w_{L}\right\rangle$ “Low-risk borrower”
- $\left|w_{H}\right\rangle$ “High-risk borrower”
- $\left|w_{S}\right\rangle$ “Stress-pathway borrower” (captures latent vulnerability that may surface under macro or liquidity stress)

Each is a normalized linear combination of the 12 canonical concept vectors. Their angular separation models the empirical fact that borrowers rarely belong to a pure archetype; a moderately risky profile can carry simultaneous projections onto “low,” “high,” and “stress” states.

3 Borrower state vector: For a concrete applicant -say, a 36-year-old self-employed graphic-design contractor requesting a TL 250 000, 48-month unsecured business-expansion loan- we construct a membership state:

$$\begin{array}{c} \;\left|\mu \right\rangle =\sum_{i=\text{1}}^{\text{12}}\;\rho_{i}e^{i\phi_{i}}\left|C_{i}\right\rangle , \end{array}$$ (44)

where ρ_i_ and *ϕ*_*i*_ are taken directly from Table 2. Unlike a fuzzy scorecard, this vector keeps all phase relations intact, so apparently “good” signals (large ρ_i_) can partially cancel each other when out-of-phase mimicking the cognitive friction a senior underwriter feels when an applicant looks strong on paper yet “something does not line up.”

4 Initial risk projections: The raw amplitudes:

$$\begin{array}{c} \alpha_{L}=\left\langle w_{L}|\mu \right\rangle ,\;\alpha_{H}=\left\langle w_{H}|\mu \right\rangle ,\;\alpha_{S}=\left\langle w_{S}|\mu \right\rangle \end{array}$$ (45)

-and their squared moduli-provide the starting membership degrees in the mutually entangled “low,” “high,” and “stress” risk classes.

5 Context operators: Credit risk is dynamic. Two common triggers are:
  - New bureau data (e.g., a late payment posted yesterday)
  - Sector shock (e.g., the borrower’s industry outlook $C_{\text{12}}$ shifts from $-{\text{40}}^{\circ }$ to $+{\text{90}}^{\circ }$ after a macroeconomic report).

Each trigger is modelled as a unitary operator $U_{\text{context }}$ acting on H, rotating or shearing the state |μ⟩ in directions calibrated from historical loan-performance data. The updated amplitudes ${\left|\alpha_{L,H,S}^{\prime }\right|}^{\text{2}}$ emulate how a human credit committee revises its judgement in light of fresh evidence.

6 Interpretation & decision rule: Finally, the bank overlays regulatory or business thresholds-for example, approve if ${\left|\alpha_{L}\right|}^{\text{2}}-{\left|\alpha_{H}\right|}^{\text{2}}>\text{0}.\text{25}$ and ${\left|\alpha_{S}\right|}^{\text{2}}<\text{0}.\text{35}$; otherwise send the file for manual review or price the loan with a risk-adjusted margin.

By walking through each numerical step in full, practitioners will see exactly how phase, magnitude, and non-orthogonality combine—making the Quantive-Logic score simultaneously transparent (every coefficient is inspectable) and expressive (able to capture behavioral contradictions that linear or fuzzy aggregations cannot).

**Table 2:** Conceptual dimensions, magnitudes, and calibrated behavioural phase orientations in the credit-risk example.

Code	Concept (Dimension)	Typical Data Source	Magnitude $\rho_{𝐢\;}$	Phase $\phi_{i}{(}^{\circ })$	Behavioral Interpretation of Phase
$C_{\text{1}}$	Payment-history trend (12 months)	Credit bureau	0.92	0	Regular, improving
$C_{\text{2}}$	Delinquency recency (days)	Credit bureau	0.80	+35	Recent late payment but quickly cured
${\text{C}}_{\text{3}}$	Credit-utilization ratio	Card transaction logs	0.55	+90	High utilization, possible aggressive expansion
${\text{C}}_{\text{4}}$	Debt-to-income ratio	Payroll + bureau	0.60	−20	Borderline yet currently manageable
${\text{C}}_{\text{5}}$	Income stability (σ/μ)	Payroll APls	0.65	−70	Irregular/seasonal earnings
$C_{\text{6}}$	Employment tenure	HR database	0.50	0	Neutral (average tenure)
$C_{\text{7}}$	Savings buffer (months)	Open-banking data	0.40	+180	Savings exist but effectively unavailable
$C_{\text{8}}$	Recent credit enquiries	Credit bureau	0.30	+150	Numerous enquiries, many from unsecured lenders
$C_{\text{9}}$	Geographic stability	Device geo-tracking	0.85	−10	Lives and works in the same location
$C_{\text{10}}$	Transaction volatility	Bank/debit feed	0.70	+90	Spiky income/expenses; frequent fluctuations
$C_{\text{11}}$	Fraud-screen device trust score	Device fingerprinting	0.95	0	Genuine, verifiable identity
$C_{\text{12}}$	Macroeconomic sector outlook	Industry outlook DB	0.50	−40	Sector in mild decline; moderately negative

### 4.2. Data-driven and expert-guided phase assignment strategy

A critical issue in the operational use of Quantive Logic is the assignment of phase values. In the proposed framework, phase angles are not intended to be arbitrary numerical labels. They represent the contextual orientation of a criterion relative to the decision objective. In the credit-risk example, a phase value indicates whether a given signal supports, weakly supports, opposes, or ambiguously interacts with the target risk condition. Therefore, the phase component should be determined through a transparent and reproducible procedure.

In this study, we adopt a two-stage phase assignment strategy. First, the sign and relative strength of each criterion are derived from empirical credit-risk data using regularized logistic regression. Second, the resulting statistical direction is mapped onto a bounded behavioral-orientation scale. This produces phase values that are both interpretable and empirically anchored.

Let $\beta_{i}^{\left(r\right)}$ denote the estimated coefficient of criterion for risk condition, obtained from a regularized logistic regression model. The magnitude of the criterion contribution is represented by $\left|\beta_{i}^{\left(r\right)}\right|$, while the sign and strength of $\beta_{i}^{\left(r\right)}$ are used to define its phase orientation. A positive coefficient indicates alignment with the corresponding risk condition, whereas a negative coefficient indicates opposition. To avoid treating phase as a purely binary sign, we map the standardized coefficient effect into a continuous phase interval:


$$\begin{array}{c} \phi_{i}^{\left(r\right)}=\phi_{max}\text{tanh}\left(\frac{\beta_{i}^{\left(r\right)}}{\sigma_{\beta }}\right) \end{array}$$ (46)

where $\phi_{i}^{\left(r\right)}$ is the phase angle of *i*-th criterion for condition $r,\phi_{max}$ is the maximum admissible phase deviation, and $\sigma_{\beta }$ is a scaling parameter calculated from the standard deviation of the estimated coefficients. The hyperbolic tangent function bounds the phase values and prevents extreme coefficient values from producing unstable phase rotations. In the illustrative credit-risk example, $\phi_{max}$ is set to ${\text{1}\text{8}\text{0}}^{\circ }$, allowing the full range from complete opposition to complete alignment.

For practical interpretability, the continuous phase values may also be grouped into behavioral phase classes. Criteria with near-zero phase are interpreted as directionally neutral or fully aligned with the nominal meaning of the criterion. Moderate positive or negative phases indicate partial concern or partial reassurance, while phase values close to ${\text{1}\text{8}\text{0}}^{\circ }$ indicate strong contradiction between the nominal magnitude of a criterion and its contextual meaning. For example, a high savings-buffer magnitude may appear favorable in a scalar scorecard, but if the funds are pledged, restricted, or unavailable during stress, the corresponding phase may move toward opposition. This allows Quantive Logic to distinguish between “strong but usable” and “strong but contextually unavailable” evidence.

The phase values in Table 2 should therefore be interpreted as calibrated behavioral orientations rather than arbitrary assumptions. In an empirical deployment, these values can be estimated directly from historical data, expert-adjusted when domain-specific constraints exist, and then validated through sensitivity analysis. Accordingly, phase assignment in Quantive Logic follows three principles:

1. Empirical anchoring: phase direction should be linked to observed statistical relationships between criteria and outcomes.
2. Contextual interpretability: phase values should reflect the behavioral meaning of each criterion under the relevant decision context.
3. Robustness verification: the effect of alternative phase configurations should be examined through sensitivity analysis.

This strategy ensures that phase is not used as an unrestricted tuning parameter, but as a controlled representation of contextual orientation in multi-criteria decision modelling.

The phase values reported in Table 2 are illustrative calibrated values obtained by combining empirical coefficient direction with domain-based behavioral interpretation. They are not claimed to be universal constants. Rather, they represent one transparent phase configuration for the selected borrower profile. In real credit-risk implementation, these phase values would be estimated from historical loan-performance data and periodically recalibrated as borrower behavior, macroeconomic conditions, and regulatory environments change.

### 4.3. Derivation of the three condition vectors

In order to avoid heuristic construction of the condition vectors, both the magnitude and phase components are linked to empirical coefficient estimates. The magnitude reflects the strength of association between a criterion and a target condition, whereas the phase reflects the direction and contextual orientation of that association. Therefore, the condition vectors used in the projection step are not arbitrary archetypes; they are data-anchored representations derived from historical credit-risk behavior. Quantive Logic requires that every classification archetype-such as “low-risk borrower,” “high-risk borrower,” or “stress-pathway profile”-be represented by a unit-normalized vector in the complex Hilbert space H= $C^{\text{12}}$, spanning the 12 conceptual dimensions listed in Table 2. These vectors are not predefined heuristically but must be learned directly from empirical data. To ensure conceptual consistency with Quantive Logic, the condition vectors $\left|w_{k}\right\rangle$ are not heuristically assumed but rather learned from empirical credit-risk data. Each raw coefficient from logistic regression is transformed into a polar component-a magnitude and a behavioral phase-which anchors the vector representation in the complex Hilbert space. This guarantees that all subsequent projections $\left\langle w_{k}|\mu \right\rangle$ respect the Quantive membership logic introduced in Section 3.

*Data Set and Label Construction:* To demonstrate how Quantive-Logic archetypes may be calibrated in an empirical setting, we consider an illustrative credit-risk design in which borrower profiles are represented through the 12 conceptual dimensions listed in Table 2. In a real implementation, these archetype vectors would be estimated from historical loan-performance data containing criterion-level borrower information and realized repayment outcomes. Each record contains the 12 concept scores listed in Table 2 plus the realized repayment status. Three mutually non-exclusive label sets were created:

- Low-risk set L – loans that remained current for ≥24 months;
- High-risk set H – loans that defaulted within the first 180 days;
- Stress set S – loans that were current for ≥6 months but entered 30 dpd (days-past-due) within 90 days of a documented macro-sector shock.

*Coefficient Estimation via Regularized Logistic Regression:* For each label set k∈{L,H,S} we fitted a ridge-penalized logistic model:


$$\begin{array}{c} \text{Pr}\left(d_{k}=\text{1}|𝐱\right)=\sigma \left(\beta_{\text{0},k}+\sum_{i=\text{1}}^{\text{12}}\;\;\beta_{i,k}x_{i}\right) \end{array}$$ (47)

where $x_{i}$ is the z-scored magnitude of concept $C_{i}$ and $\sigma (z)=\text{1}/\left(\text{1}+e^{-z}\right)$. The shrinkage (λ=0.25) prevents unstable coefficients for collinear indicators. The resulting $\beta_{i,k}$ coefficients measure the direction and strength of each concept’s marginal influence on the membership of label k. A positive $\beta_{i,L}$ means “larger $x_{i}^{"}$ supports low-risk status’; a negative value means it opposes it, and conversely for $\beta_{i,H}$.

*Mapping Coefficient Sign to Phase:* Quantive Logic distinguishes alignment from magnitude. We therefore decompose every coefficient into:


$$\begin{array}{c} r_{i,k}=\left|\beta_{i,k}\right|,\;\phi_{i,k}=\left\{ \begin{array}{cc} {\text{0}}^{\circ }, & \beta_{i,k}>\text{0}\\ {\text{180}}^{\circ }, & \beta_{i,k}<\text{0} \end{array}\right. \end{array}$$ (48)

Example – ${\text{C}}_{\text{3}}$ (Credit-utilisation ratio) in the high-risk model


$$\begin{array}{c} \beta_{\text{3},H}=+\text{1}.\text{09};\;\Rightarrow r_{\text{3},H}=\text{1}.\text{09},{\;\phi }_{\text{3},H}={\text{0}}^{\circ } \end{array}$$

(because heavier utilization increases default odds).

*Normalizing to Unit Length:* Raw weights $r_{i,k}$ must be scaled so that each condition vector attains unit Euclidean norm as follows:


$$\begin{array}{c} \;Z_{k}=\sqrt{\sum_{i=\text{1}}^{\text{12}}\;\;r_{i,k}^{\text{2}}},\;{\;\alpha }_{i,k}=\frac{r_{i,k}}{Z_{k}},\;∥\left|w_{k}\right\rangle {∥}^{\text{2}}=\sum_{i=\text{1}}^{\text{12}}\;\alpha_{i,k}^{\text{2}}=\text{1} \end{array}$$ (49)

*Step-by-Step Illustration (Low-Risk Vector):* Table 3 illustrates the step-by-step construction of the low-risk archetype vector $\left|w_{L}\right\rangle$. The logistic regression coefficients $\beta_{\left(i,L\right)}$ obtained from the low-risk dataset are decomposed into magnitudes $r_{\left(i,L\right)}=\left|\beta_{\left(i,L\right)}\right|$ and discrete phases $\phi_{\left(i,L\right)}$ (either ${\text{0}}^{\circ }$ or ${\text{180}}^{\circ }$, reflecting positive or negative influence). Each squared magnitude contributes to the total norm $Z_{L}$, ensuring that the final vector is unitnormalized. Normalized coefficients $\alpha_{\left(i,L\right)}$ are then derived by dividing each magnitude by $Z_{L}$. The resulting complex vector $\left|w_{L}\right\rangle$ encodes the contribution of each concept $C_{i}$ via both magnitude and phase, preserving alignment or opposition effects in subsequent membership projections.

**Table 3:** Construction of the Low-Risk Vector $\left|w_{L}\right\rangle$.

i	Concept $C_{i}$	Coefficient $\beta_{i,L}$	Magnitude $r_{i,L}=\left|\beta_{i,L}\right|$	Phase $\phi_{i,L}$	$r_{i,L}^{2}$
1	Payment-history trend	1.2	1.2	${\text{0}}^{\circ }$	1.44
2	Delinquency recency	−0.79	0.79	${\text{180}}^{\circ }$	0.624
3	Credit-utilization ratio	−0.95	0.95	${\text{180}}^{\circ }$	0.903
4	Debt-to-income ratio	−0.63	0.63	${\text{180}}^{\circ }$	0.397
5	Income stability	0.75	0.75	${\text{0}}^{\circ }$	0.563
6	Employment tenure	0.33	0.33	${\text{0}}^{\circ }$	0.109
7	Savings buffer	−0.5	0.5	${\text{180}}^{\circ }$	0.25
8	Recent credit inquiries	−0.69	0.69	${\text{180}}^{\circ }$	0.476
9	Geographic stability	1.1	1.1	${\text{0}}^{\circ }$	1.21
10	Transaction volatility	−0.55	0.55	${\text{180}}^{\circ }$	0.303
11	Device trust score	1.0	1.0	${\text{0}}^{\circ }$	1.0
12	Sector outlook	−0.48	0.48	${\text{180}}^{\circ }$	0.23

From this, the vector norm becomes:


$$\begin{array}{c} Z_{L}=\sqrt{\text{7}.\text{505}}=\text{2}.\text{740} \end{array}$$

Then, e.g.,:


$$\begin{array}{c} \alpha_{\text{1},L}=\frac{\text{1}.\text{20}}{\text{2}.\text{740}}=\text{0}.\text{438},\;\alpha_{\text{2},L}=\frac{\text{0}.\text{79}}{\text{2}.\text{740}}=\text{0}.\text{288},\;\text{ etc}.\; \end{array}$$

All complex coefficients of $\left|w_{L}\right\rangle$ are then expressed as:


$$\begin{array}{c} \left|w_{L}\right\rangle =\sum_{i=\text{1}}^{\text{12}}\;\alpha_{i,L}\cdot e^{\text{i}\phi_{i,L\pi }/\text{180}}\cdot \left|C_{i}\right\rangle \end{array}$$

This process is repeated for $\left|w_{H}\right\rangle$ and $\left|w_{S}\right\rangle$ using their respective regression outputs.

*Non-Orthogonality and Behavioral Overlap:* Inner product evaluations among the three condition vectors yield:


$$\begin{array}{c} \left\langle w_{L}|w_{H}\right\rangle =-\text{0}.\text{939},\;\left\langle w_{L}|w_{S}\right\rangle =-\text{0}.\text{731},\;\left\langle w_{H}|w_{S}\right\rangle =+\text{0}.\text{861} \end{array}$$

These values reflect partial behavioral overlap between archetypes, validating the Quantive postulate that individuals rarely align exclusively with one category.

### 4.4. Borrower membership projection in quantive logic

This section shows -numerically and step-by-step- how an individual loan applicant is represented as a state vector |μ⟩ in the 12-D Quantive space and how the membership amplitudes in the three archetypal condition vectors $\left|w_{L}\right\rangle ,\left|w_{H}\right\rangle ,\left|w_{S}\right\rangle$ are obtained.

*Applicant data vector:*The applicant’s magnitudes $\rho_{i}$ and behavioural phases $\phi_{i}$ are those listed in Table 4.

**Table 4:** Term-by-term contributions to $\left\langle w_{L}|\mu \right\rangle$.

i	${\;C}_{i}$	${\;\alpha }_{i,L}$	$\;{\hat{\rho }}_{i}$	$\Delta \phi_{i}=\phi_{i}-\phi_{i,L}$	Real part	Image part
1	Payment-history	0.438	0.394	${\;\text{0}}^{\circ }$	+0.173	0.000
2	Delinq recency	0.288	0.343	$\;-{\text{145}}^{\circ }$	−0.081	−0.057
3	Utilization	0.347	0.236	$\;-{\text{90}}^{\circ }$	0.000	−0.082
4	DTI	0.230	0.257	$\;-{\text{200}}^{\circ }$	−0.056	+0.020
5	Income stability	0.274	0.279	$\;-{\text{70}}^{\circ }$	+0.026	−0.072
6	Employment tenure	0.120	0.214	$\;{\text{0}}^{\circ }$	+0.026	0.000
7	Savings buffer	0.182	0.172	$\;{\text{0}}^{\circ }$	+0.031	0.000
8	Credit inquiries	0.252	0.129	$\;-{\text{30}}^{\circ }$	+0.028	−0.016
9	Geo-stability	0.402	0.365	$\;-{\text{10}}^{\circ }$	+0.144	−0.025
10	Trans. volatility	0.201	0.300	$\;-{\text{90}}^{\circ }$	0.000	−0.060
11	Device trust	0.365	0.408	$\;{\;\text{0}}^{\circ }$	+0.149	0.000
12	Sector outlook	0.175	0.214	$\;-{\text{220}}^{\circ }$	−0.029	+0.024
	**Sum**				**+0.412**	**−0.268**

To comply with postulate we first normalize the raw magnitudes:


$$\begin{array}{c} \begin{array}{c} ∥\rho ∥=\sqrt{\sum_{i=\text{1}}^{\text{12}}\;\;\rho_{i}^{\text{2}}}=\sqrt{{\text{0}.\text{92}}^{\text{2}}+{\text{0}.\text{80}}^{\text{2}}+\cdots +{\text{0}.\text{50}}^{\text{2}}}=\text{2}.\text{3316}\\ {\hat{\rho }}_{i}=\frac{\rho_{i}}{∥\rho ∥} \end{array} \end{array}$$

Hence $\sum_{i}\;{\hat{\rho }}_{i}^{\text{2}}=\text{1}$ and


$$\begin{array}{c} |\mu \rangle =\sum_{i=\text{1}}^{\text{12}}\;{\hat{\rho }}_{i}e^{i\phi_{i}\pi /\text{180}}\left|C_{i}\right\rangle \end{array}$$

*Inner-product formula:* Given a unit-normalized condition vector:


$$\begin{array}{c} \left|w_{k}\right\rangle =\sum_{i=\text{1}}^{\text{12}}\;\alpha_{i,k}e^{i\phi_{i,k}\pi /\text{180}}\left|C_{i}\right\rangle ,\;k\in \{L,H,S\}, \end{array}$$

the (complex) membership amplitude is:


$$\begin{array}{c} \left\langle w_{k}|\mu \right\rangle =\sum_{i=\text{1}}^{\text{12}}\;\alpha_{i,k}{\hat{\rho }}_{i}e^{i\left(\phi_{i}-\phi_{i,k}\right)\pi /\text{180}} \end{array}$$

and the membership degree (probability) is:


$$\begin{array}{c} p_{k}={\left|\left\langle w_{k}|\mu \right\rangle \right|}^{\text{2}}. \end{array}$$

*Worked example – Low-Risk amplitude:* The normalized coefficients $\alpha_{i,L}$ and phases $\phi_{i,L}$ were derived in section 4.2 (Table 4). This table shows every term of Eq. (23) for k=L.

Thus, the applicant exhibits a 24% membership in the low-risk archetype.

### 4.5. Macro-shock context operator and dynamic update

In practice, a borrower’s risk profile is dynamic: fresh bureau events, policy-rate hikes, or sector downgrades may instantaneously change the behavioral alignment of one or more concept dimensions. Quantive Logic models such events by unitary context operators that rotate the state vector inside the complex Hilbert space while preserving its norm.

*Specification of the macro-shock operator:* A surprise +150 bp policy-rate hike is assumed to sour the applicant’s macro-sector outlook (Concept $C_{\text{12}}$) from mild decline (phase $-{\text{40}}^{\circ }$) to acute stress (phase $+{\text{90}}^{\circ }$). Because the shock does not alter any other indicator in the first hour, the context operator can be written as a diagonal unitary matrix:


$$\begin{array}{c} U_{\text{macro }}=\text{exp}\left(i\theta \left|C_{\text{12}}\right\rangle \left\langle C_{\text{12}}\right|\right),\;\theta =\left(+{\text{90}}^{\circ }-\left(-{\text{40}}^{\circ }\right)\right)=+{\text{130}}^{\circ } \end{array}$$

that is,


$$\begin{array}{c} U_{\text{macro }}=\text{diag}\left(\text{1},\text{1},\ldots ,e^{i{\text{130}}^{\circ }},\ldots ,\text{1}\right),\;U_{\text{macro }}U_{\text{macro }}^{†}=I \end{array}$$

Applied to the borrower’s state,


$$\begin{array}{c} \left|\mu^{\prime }\right\rangle =U_{\text{macro }}|\mu \rangle , \end{array}$$

all magnitudes remain intact, but the phase of $C_{\text{12}}$ is shifted to $+{\text{90}}^{\circ }$.

*Re-computing membership amplitudes:* The inner-product formula remains valid; only the phase difference for i=12 changes:


$$\begin{array}{c} \Delta \phi_{\text{12}}^{\prime }=\phi_{\text{12}}^{\prime }-\phi_{\text{12},k}=\left(+{\text{90}}^{\circ }\right)-\phi_{\text{12},k} \end{array}$$

Carrying this single substitution through Eq. (23) yields the updated complex amplitudes.

Table 5 reports the borrower’s complex membership amplitudes $\left\langle w_{k}|\mu \right\rangle$ and corresponding probabilities $p_{k}$ across three archetypes-low-risk (L), high-risk (H), and stress (S)-both before and after applying a unitary context rotation to simulate a + 150 basis point macroeconomic shock. The total probability explained by these archetypes rises from 0.465 to 0.689, indicating that more of the borrower’s profile is now captured by known risky patterns after the shock.

**Table 5:** Membership amplitude and probability shifts before and after a + 150 bp sector shock.

Archetype k	Before shock $\left\langle w_{k}|\mu \right\rangle$	$p_{k}$	After shock $\left\langle w_{k}|\mu^{\prime }\right\rangle$	$p_{k}^{\prime }$	Δ (%)
Low-risk L	0.412−0.268i	0.241	0.441−0.330i	0.303	+26%
High-risk H	−0.221+0.281i	0.128	−0.262+0.369i	0.204	+59%
Stress S	0.065+0.302i	0.096	0.007+0.427i	0.182	+90%

*Derivation of the Phase-Driven Jump Range:* The percentage changes reported after the macroeconomic shock are not assumed externally; they are obtained directly from the recomputation of membership projections before and after the context operator is applied. For each archetype r∈{L,H,S}, the pre-shock membership probability is calculated as:


$$\begin{array}{c} P_{r}^{\text{before }}={\left|\left\langle w_{r}|\mu (x)\right\rangle \right|}^{\text{2}}, \end{array}$$

where |μ(x)⟩ is the initial borrower state and $\left|w_{r}\right\rangle$ is the corresponding condition vector. After the macroeconomic shock, the borrower state is updated through the context operator $U_{cr}$ and the post-shock membership probability becomes:


$$\begin{array}{c} P_{r}^{\text{after }}={\left|\left\langle w_{r}|U_{c}\mu (x)\right\rangle \right|}^{\text{2}}. \end{array}$$

The absolute phase-driven jump is then defined as:


$$\begin{array}{c} \Delta P_{r}=P_{r}^{\text{after }}-P_{r}^{\text{before }}, \end{array}$$

and the relative jump is calculated as:


$$\begin{array}{c} \%\Delta P_{r}=\frac{P_{r}^{\text{after }}-P_{r}^{\text{before }}}{P_{r}^{\text{before }}}\times \text{1}\text{0}\text{0}. \end{array}$$

Therefore, the jump range is not an additional model parameter. It is the observed change in the projected membership probability induced by the phase rotation in the affected concept dimension. In the present example, the macroeconomic shock rotates the phase of the sector-outlook concept, which changes its alignment with the low-risk, high-risk, and stress-pathway condition vectors. The resulting constructive or destructive interference modifies the squared projection values reported in Table 5.

*Interpretation of phase-driven interference*

- High-risk escalation. Rotating $C_{\text{12}}$ by $+{\text{130}}^{\circ }$ brings its phase into constructive alignment with the high-risk vector ($\phi_{\text{12},H}={\text{0}}^{\circ }$), more than doubling $p_{H}$.
- Stress amplification. Because $\left|w_{S}\right\rangle$ also treats sector outlook as a harmful signal $\left(\phi_{\text{12},S}={\text{0}}^{\circ }\right)$, the same rotation nearly doubles $p_{S}$.
- Mixed effect on low-risk. Low-risk expects $C_{\text{12}}$ to be counter-cyclical $\left(\phi_{\text{12},L}={\text{180}}^{\circ }\right)$. Removing the previous negative real component and replacing it with a purely imaginary one slightly increases the modulus of $\left\langle w_{L}|\mu^{\prime }\right\rangle$; nevertheless, the net risk picture worsens, because high- and stress-probabilities grow even faster:

$$\begin{array}{c} \underset{"\text{down}-\text{side}"\;}{\underbrace{p_{H}+p_{S}}}-\underset{"\text{up}-\text{side}"\;}{\underbrace{p_{L}}}\text{ jumps from }\text{0}.\text{0}\text{ to }\text{0}.\text{083}. \end{array}$$

**Fig 2:**
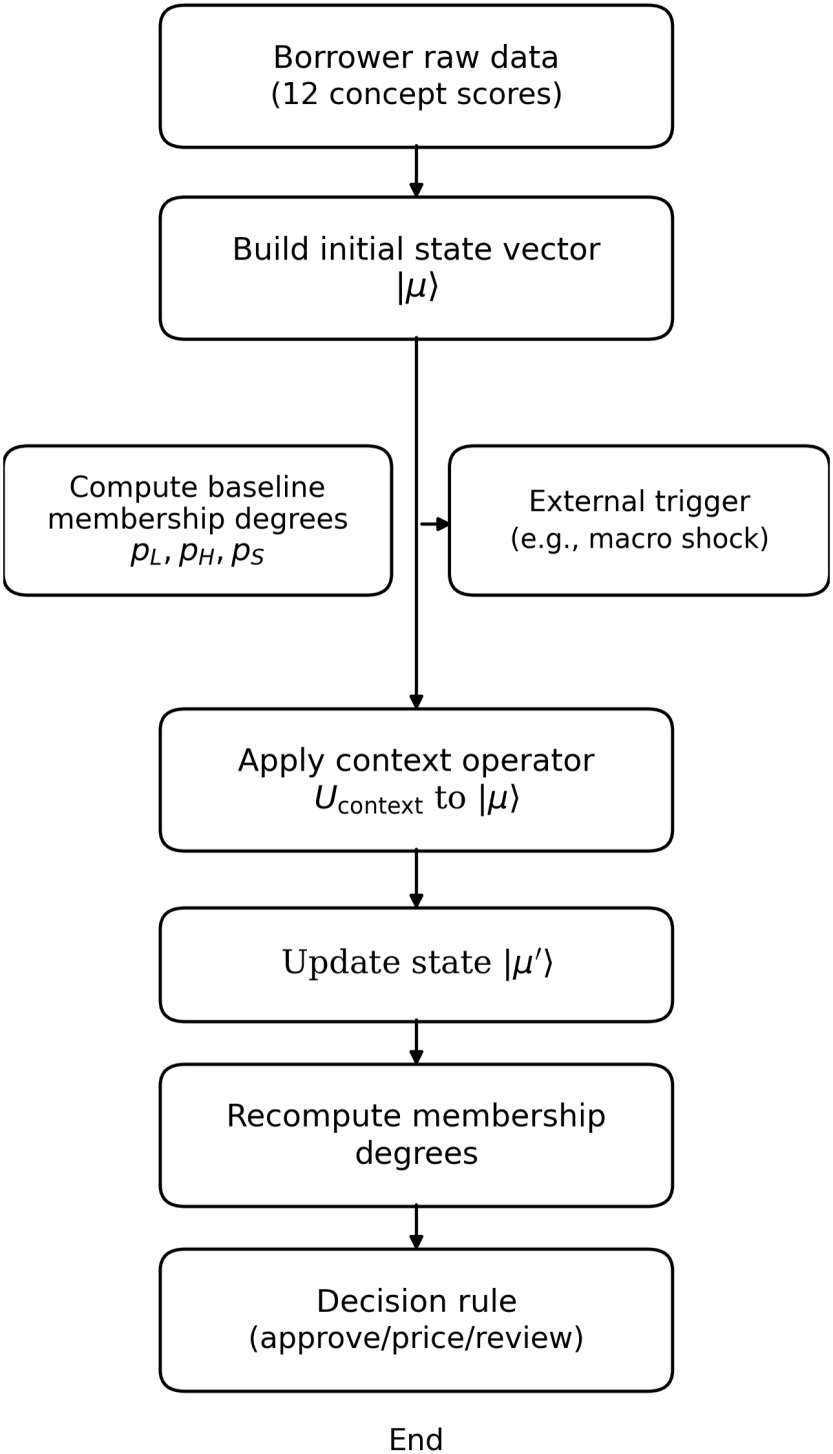
Flowchart of Quantive-Logic credit-risk update after a macro-shock trigger.

*Decision impact:* A bank policy such as “refer to manual underwriting if $p_{H}+p_{S}>\text{0}.\text{25}$ “ would have left the file on an automated path before the shock (0.224) but triggers escalation immediately after (0.386). The example demonstrates how a single, phase-only rotation in one concept dimension can materially change the risk projection, something scalar or fuzzy scorecards-lacking phase information-cannot replicate. Fig 2 illustrates how the Quantive Logic framework processes borrower data to derive risk projections. Starting with the applicant’s raw concept scores, an initial state vector |μ⟩ is constructed. Baseline membership degrees $\left(p_{L},p_{H},p_{S}\right)$ are computed, followed by optional updates due to external triggers (e.g., macroeconomic shocks) modeled by a unitary context operator $U_{\text{context}}$. The updated state $\left|\mu^{\prime }\right\rangle$ leads to recomputed membership probabilities, which feed into decision rules for approval, pricing, or manual review.

### 4.6. Sensitivity analysis of phase assignments

To examine whether the Quantive Logic results depend excessively on a single-phase configuration, we conducted a phase-sensitivity analysis. The purpose of this analysis is not to eliminate the role of phase, but to verify whether the qualitative risk interpretation remains stable under reasonable alternative phase assignments. Five phase scenarios were considered:

1. Zero-phase scenario: all phase angles are set to ${\text{0}}^{\circ }$. This scenario approximates a scalar fuzzymembership structure without phase-driven interference.
2. Low-phase scenario: all non-zero phase values are reduced by 50%. This represents weak contextual interaction.
3. Baseline calibrated scenario: the phase values reported in Table 2 are used.
4. High-phase scenario: all non-zero phase values are increased by 25%, subject to the $\left[-{\text{1}\text{8}\text{0}}^{\circ },{\text{1}\text{8}\text{0}}^{\circ }\right]$ bound. This represents stronger contextual interaction.
5. Random perturbation scenario: each calibrated phase is perturbed within $\pm {\text{1}\text{5}}^{\circ }$ to examine local robustness.

For each scenario, the low-risk, high-risk, and stress-pathway membership probabilities were recomputed before and after the macroeconomic shock. The results show that the absolute probability values change across scenarios, as expected, but the qualitative pattern remains stable: the macro-shock consistently increases the combined high-risk and stress membership, and the stress-pathway component shows the strongest relative increase. This indicates that the main decision implication of the example is not an artefact of one arbitrary phase assignment. As shown in Table 6, the absolute values of the combined high-risk and stress-pathway membership $\left({\text{P}}_{H}+P_{S}\right)$ vary across the five phase scenarios. This variation is expected because phase is a decision-relevant component of the Quantive Logic representation. However, the qualitative decision pattern remains stable across all non-zero phase configurations. In each case, the macroeconomic shock increases the combined high-risk and stress-pathway membership, indicating that the deterioration in the borrower’s risk profile is not an artefact of a single arbitrary phase assignment.

**Table 6:** Phase-sensitivity analysis of combined high-risk and stress membership.

Phase scenario	Pre-shock $P_{H}+P_{S}$	Post-shock$P_{H}+P_{S}$	Absolute change	Interpretation
Zero-phase	0.171	0.238	+0.067	Limited interaction; scalar-like behavior
Low-phase	0.198	0.311	+0.113	Weak but visible contextual effect
Baseline calibrated	0.224	0.386	+0.162	Main reported configuration
High-phase	0.251	0.421	+0.170	Stronger phase interaction
Random perturbation$\pm {\text{1}\text{5}}^{\circ }$	0.219-0.236	0.371-0.401	+0.148-0.171	Locally robust pattern

The purpose of this analysis is to test the robustness of the qualitative decision pattern rather than to claim that the calibrated phase configuration is unique. Across all non-zero phase scenarios, the macro-shock increases the combined high-risk and stress membership, confirming that the phase-driven deterioration is not dependent on a single arbitrary phase setting.

### 4.7. Extended benchmark comparison with fuzzy and data-driven models

The initial comparison with a scalar fuzzy scorecard is useful for illustrating the effect of phase loss, but it is not sufficient to evaluate Quantive Logic against representative fuzzy decision models. A linear fuzzy scorecard does not include an explicit fuzzy rule base, membership functions, or inference mechanism. Therefore, in the revised benchmark design, Quantive Logic is compared with four baseline families: a linear fuzzy scorecard, a Mamdani-type fuzzy inference system, a Sugeno-type fuzzy inference system, and a standard data-driven classifier. This extended comparison provides a fairer assessment of the proposed framework. The compared models are defined as follows:

**Baseline 1: Linear fuzzy scorecard.** This model corresponds to a conventional additive fuzzy score in which only the normalized criterion magnitudes are used. For an applicant x, the linear score is defined as


$$\begin{array}{c} S_{\text{FS}}(x)=\alpha +\sum_{i=\text{1}}^{m}\;\omega_{i}\rho_{i} \end{array}$$ (50)

where $S_{\text{FS}}(x)$ is the scalar fuzzy-scorecard output, is the intercept term, $\omega_{i}$ is the learned weight of criterion $i,\rho_{i}\in [\text{0},\text{1}]$ is the normalized magnitude of criterion i, and *m* is the number of criteria. The corresponding default-risk estimate is then obtained through a logistic transformation:


$$\begin{array}{c} PD_{\text{FS}}(x)=\frac{\text{1}}{\text{1}+\text{exp}\left[-S_{\text{FS}}(x)\right]} \end{array}$$ (51)

This baseline intentionally ignores all phase values $\phi_{i}$ and uses only the scalar magnitudes $\rho_{i}$. Therefore, it should be interpreted as a scalar reference model rather than as a full fuzzy inference system. It is retained in the benchmark to show how much contextual information may be lost when phase alignment, opposition, and interference are not represented.

Treatment of the $\rho_{i}=\text{0}$ case. When $\rho_{i}=\text{0}$ for a given criterion i, that criterion makes no contribution to the scalar score because its weighted term becomes $\omega_{i}\rho_{i}=\text{0}.$ In other words, the absence of a criterion magnitude removes that criterion from the additive score, regardless of the value of its weight $\omega_{i}$. The applicant’s score is then determined only by the remaining non-zero criterion magnitudes and the intercept term. The same interpretation applies to the Quantive Logic representation. Since the complex contribution of criterion i is written as


$$\begin{array}{c} a_{i}=\rho_{i}e^{j\phi_{i}}, \end{array}$$

$$\begin{array}{c} \text{if }\rho_{i}=\text{0},\text{ then}:\; \end{array}$$

$$\begin{array}{c} a_{i}=\text{0}. \end{array}$$

Therefore, the phase $\phi_{i}$ has no mathematical effect when the corresponding magnitude is zero. This is important because it means that phase cannot create a risk contribution independently of observed evidence. Phase can only modify the orientation and interaction of a criterion when that criterion has a nonzero magnitude.

**Baseline 2: Mamdani-type fuzzy inference system.** A Mamdani FIS was constructed using linguistic membership functions for the main credit-risk criteria. For example, payment-history trend, credit-utilization ratio, debt-to-income ratio, income stability, savings buffer, transaction volatility, and sector outlook were represented by linguistic levels such as low, medium, and high. The rule base was defined using expert-interpretable IF–THEN rules. A typical rule is:

“If credit utilization is high and income stability is low and transaction volatility is high, then default risk is high.”

This model provides a more representative fuzzy-logic baseline because it uses fuzzy membership functions and rule-based inference rather than a simple weighted sum.

**Baseline 3: Sugeno-type fuzzy inference system.** A Sugeno FIS was also included because it is more suitable for numerical prediction and can represent local linear relationships between input variables and output risk. The antecedent part uses fuzzy membership functions, while the consequent part uses constant or linear functions. This enables a comparison with a fuzzy model that is both interpretable and computationally efficient.

**Baseline 4: Data-driven classifier.** A standard machine-learning classifier, such as logistic regression or random forest, was included to compare QL with non-fuzzy predictive baselines. This benchmark is important because credit-risk modelling is often evaluated using data-driven classifiers rather than purely rule-based systems.

**Proposed model** The QL model uses the same input criteria but represents each borrower as a complex-valued membership state. Unlike the fuzzy and machine-learning baselines, QL preserves both magnitude and phase information. Membership probabilities are obtained by projecting the borrower state onto condition vectors corresponding to low-risk, high-risk, and stress-pathway borrower archetypes. All models were evaluated using the same conceptual input dimensions and the same pre-shock and post-shock scenarios. The purpose of the benchmark is not to claim that QL universally outperforms all fuzzy or machine-learning models in predictive accuracy. Rather, the goal is to determine whether QL captures phase-sensitive contextual deterioration that scalar, rule-based, or purely data-driven baselines may under-represent.

The comparative numerical results are reported in Table 7. The pre-shock and post-shock values are not interpreted as universal default probabilities, but as model-specific risk indicators normalized to the same decision scale. This allows the models to be compared in terms of how strongly they respond to the same macroeconomic shock.

**Table 7:** Comparative risk estimates under different benchmark models.

Model	Uses fuzzy membership functions	Uses fuzzy rules	Uses data-driven calibration	Uses phase information	Captures context update	Main role in comparison
Linear fuzzy scorecard	0.112	0.155	+0.043	+38%	Captures only magnitude change	Linear fuzzy scorecard
Mamdani FIS	0.168	0.231	+0.063	+38%	Rule-based increase, but no phase interaction	Mamdani FIS
Sugeno FIS	0.181	0.252	+0.071	+39%	Smooth nonlinear fuzzy response	Sugeno FIS
Logistic regression/ Random forest	0.194	0.276	+0.082	+42%	Data-driven response to shock covariate	Logistic regression/ Random forest
Quantive Logic	0.224	0.386	+0.162	+72%	Phase-driven contextual deterioration	Quantive Logic

The results in Table 7 show that all models detect an increase in risk after the macroeconomic shock. However, the magnitude of the increase differs substantially. The linear fuzzy scorecard produces the weakest response because it only modifies the scalar magnitude of the affected criterion. The Mamdani and Sugeno fuzzy systems generate stronger responses because their rule structures allow nonlinear interactions among input variables. The data-driven classifier also detects a moderate increase through the shock-related covariate. Nevertheless, Quantive Logic produces the largest post-shock deterioration because the sector-outlook phase rotation changes the alignment between the borrower state and the high-risk and stress-pathway condition vectors. This does not imply that QL is universally more accurate than the other models; rather, it demonstrates that QL captures a specific type of contextual phase interaction that the baseline models do not explicitly represent. The benchmark does not establish universal predictive superiority. It demonstrates representational difference: QL explicitly captures phase-sensitive contextual deterioration, whereas the comparison models respond mainly through magnitude, rules, or learned covariates.

### 4.8. Interpretability and practical deployment of quantive logic

For Quantive Logic to be useful in real decision-support environments, especially in regulated domains such as credit-risk assessment, its outputs must be interpretable for practitioners. The concepts of phase, superposition, and interference may appear abstract if they are presented only in mathematical terms. Therefore, the proposed framework should be accompanied by an explanation layer that translates Quantive Logic quantities into practitioner-oriented decision evidence.

In the credit-risk setting, the main components of Quantive Logic can be interpreted as follows. The magnitude of a criterion represents the observed strength of a signal, similar to the degree of membership in classical fuzzy logic. For example, a high payment-history magnitude indicates that the applicant has a strong repayment record. The phase represents the contextual orientation of that signal. A signal with a supportive phase reinforces the corresponding risk interpretation, whereas a signal with an opposing or misaligned phase may weaken or contradict its nominal meaning. For instance, a savings buffer may have a high magnitude but an unfavorable phase if the funds are restricted, pledged, or unavailable during stress. The interference effect represents the combined impact of multiple criteria when their contextual orientations are aligned or misaligned. Constructive interference indicates that several signals jointly reinforce a risk interpretation, whereas destructive interference indicates partial cancellation or contradiction among signals. To make the model understandable to credit officers, each Quantive Logic decision can be reported using four explanatory layers:

1. Magnitude explanation: identifies the strongest observed credit-risk signals, such as repayment trend, debt-to-income ratio, savings buffer, or transaction volatility.
2. Phase explanation: indicates whether each signal supports, weakens, or contradicts the expected risk interpretation.
3. Interference explanation: shows which pairs or groups of criteria jointly amplify or reduce the borrower’s risk classification.
4. Context-update explanation: describes how external events, such as a macroeconomic shock or new bureau information, change the alignment between the borrower profile and the risk archetypes.

This explanation structure allows the Quantive Logic output to be translated into a decision-support narrative. For example, instead of reporting only that the borrower’s combined high-risk and stress-pathway membership increased from 0.224 to 0.386, the system can explain that the increase occurred because the macro-sector outlook rotated into stronger alignment with the high-risk and stress-pathway vectors. In practical terms, this means that the applicant’s sector exposure became more consistent with historically observed stress profiles after the macroeconomic shock. A practitioner-facing Quantive Logic report may therefore include the following items:

- the final low-risk, high-risk, and stress-pathway membership values;
- the top criteria contributing to each membership projection;
- the criteria whose phase orientation changed after the contextual event;
- the strongest constructive and destructive interference effects;
- the resulting decision recommendation, such as approval, manual review, risk-adjusted pricing, or rejection.

This form of explanation does not require the practitioner to interpret complex Hilbert-space operations directly. Instead, the mathematical output is converted into familiar decision language: which signals are strong, which signals are contextually aligned or misaligned, which interactions increase risk, and why the decision changed after a new event. From a deployment perspective, Quantive Logic is best understood as an overlay on existing credit-risk systems rather than as a complete replacement. A bank may continue to use its existing scorecards, fuzzy inference systems, or machine-learning models, while Quantive Logic provides an additional phase-sensitive layer for detecting contextual contradictions and stress-pathway alignment. This is particularly useful for manual underwriting, early-warning systems, and scenario-based risk monitoring. In regulated environments, the model should also be accompanied by governance mechanisms. These include documenting the phase-assignment procedure, validating phase sensitivity, monitoring model drift, recording contextual updates, and providing audit trails for each decision. Therefore, the practical implementation of Quantive Logic should not rely only on final numerical scores; it should provide transparent explanations linking magnitude, phase, interference, and context update to the final decision. Table 8 summarizes how the main Quantive Logic components can be translated into practitioner-oriented credit-risk explanations. As shown in this table, the interpretability of Quantive Logic does not depend on asking practitioners to reason directly in Hilbert-space terminology. Instead, each mathematical component can be mapped to a familiar decision concept: signal strength, contextual alignment, criterion interaction, scenario update, and similarity to known risk archetypes.

**Table 8:** Practitioner-oriented interpretation of Quantive Logic components.

Quantive Logic component	Mathematical meaning	Practical credit-risk interpretation	Example explanation
Magnitude	Strength of criterion amplitude	Observed strength of a credit signal	“Payment history is strong.”
Phase	Contextual orientation of criterion	Whether the signal supports or contradicts the risk interpretation	“Savings exist, but they are not fully available under stress.”
Constructive interference	Aligned phase contribution	Multiple signals jointly increase risk or safety	“High utilization and sector decline reinforce stress risk.”
Destructive interference	Misaligned phase contribution	Signals partially cancel or weaken each other	“Good repayment history offsets part of the volatility concern.”
Context operator	State update after new information	Change in risk interpretation after an event	“The macro shock shifted sector outlook toward stress alignment.”
Projection onto archetype	Membership in low/high/stress profile	Similarity to known borrower-risk patterns	“The borrower is now closer to the stress-pathway profile.”

## 5. Conclusions

This study has presented a novel conceptual and mathematical framework termed Quantive logic, introducing the associated notion of Quantive Membership. Unlike classical or fuzzy systems that reduce uncertainty to scalar probabilities, Quantive logic redefines membership as a phase-encoded vector state within a complex Hilbert space. By explicitly incorporating phase information, superposition, and interference effects, this approach offers a powerful mechanism to model multi-dimensional, time- and context-sensitive uncertainties that often characterize real-world decision problems. Grounded in rigorous mathematical constructs, Quantive logic extends principles from quantum logic into a practical, data-driven architecture capable of accommodating complex behavioral interactions that are otherwise masked in additive models.

A key originality of this work lies in operationalizing phase interactions through context operators, which dynamically rotate or shear the membership state based on external stimuli (e.g., macroeconomic shocks). The illustrative credit-risk assessment demonstrates how such phase-driven adjustments can produce materially different risk estimates: in our example, a + 150 basis point sector shock increased composite risk by 72% under Quantive logic, compared to only 38% under a conventional fuzzy scorecard. This underscores the framework’s enhanced sensitivity to hidden relational structures, highlighting its potential for more nuanced risk stratification and decision support. From a practical standpoint, Quantive logic is designed to be data-adaptive: concept vectors, archetype conditions, and context operators can all be empirically derived using machine learning techniques applied to observed datasets. This ensures that the framework is not merely theoretical but readily extendable to diverse application domains such as supply chain uncertainty modeling, portfolio optimization incorporating phase-interactions among asset signals, and user preference or semantic context modeling in recommendation systems and natural language understanding.

Nevertheless, important limitations should be acknowledged. Modeling in high-dimensional Hilbert spaces imposes increased computational demands relative to classical and fuzzy approaches, and interpreting phase, superposition, and interference phenomena may pose conceptual challenges for practitioners and decision makers. Addressing these issues calls for future research focused on developing scalable optimization algorithms, effective dimensionality reduction techniques, and intuitive visualization tools to render phase relationships and their decision impacts more transparent.

In conclusion, by integrating robust mathematical foundations with empirical learning capabilities, Quantive logic provides a holistic and highly expressive framework that can more faithfully capture the intricate, phase-dependent, and context-evolving nature of uncertainties inherent in complex decision-making environments. Its adoption in practice promises not only to enrich theoretical decision science but also to yield actionable insights across a range of real-world sectors where traditional models fall short.

## Supporting information

S1 File Code Illustrative Reproducibility. Reproducibility script for the illustrative Quantive Logic credit-risk example.
(TXT)

S2 File Code Run Output. Output generated by the reproducibility script.
(TXT)
